# Predictis: an IoT and machine learning-based system to predict risk level of cardio-vascular diseases

**DOI:** 10.1186/s12913-023-09104-4

**Published:** 2023-02-20

**Authors:** Muhammad Nazrul Islam, Kazi Rafid Raiyan, Shutonu Mitra, M. M. Rushadul Mannan, Tasfia Tasnim, Asima Oshin Putul, Angshu Bikash Mandol

**Affiliations:** Department of Computer Science and Engineering, Military Institute of Science and Technology, Dhaka-1216, Bangladesh

**Keywords:** Cardio-vascular disease, Internet of Things, Machine learning, Stacking classifier, Prediction, Risk level

## Abstract

**Background:**

Despite technological advancement in the field of healthcare, the worldwide burden of illness caused by cardio-vascular diseases (CVDs) is rising, owing mostly to a sharp increase in developing nations that are undergoing fast health transitions. People have been experimenting with techniques to extend their lives since ancient times. Despite this, technology is still a long way from attaining the aim of lowering mortality rates.

**Methods:**

From methodological perspective, a design Science Research (DSR) approach is adopted in this research. As such, to investigate the current healthcare and interaction systems created for predicting cardiac disease for patients, we first analyzed the body of existing literature. After that, a conceptual framework of the system was designed using the gathered requirements. Based on the conceptual framework, the development of different components of the system was completed. Finally, the evaluation study procedure was developed taking into account the effectiveness, usability and efficiency of the developed system.

**Results:**

To attain the objectives, we proposed a system consisting of a wearable device and mobile application, which allows the users to know their risk levels of having CVDs in the future. The Internet of Things (IoT) and Machine Learning (ML) techniques were adopted to develop the system that can classify its users into three risk levels (high, moderate and low risk of having CVD) with an F1 score of 80.4% and two risk levels (high and low risk of having CVD) with an F1 score of 91%. The stacking classifier incorporating best-performing ML algorithms was used for predicting the risk levels of the end-users utilizing the UCI Repository dataset.

**Conclusion:**

The resultant system allows the users to check and monitor their possibility of having CVD in near future using real-time data. Also, the system was evaluated from the Human-Computer Interaction (HCI) point of view. Thus, the created system offers a promising resolution to the current biomedical sector.

**Trial Registration:**

Not Applicable.

## Introduction

With the help of digitization and digital transformation, the healthcare sector has changed over the past decade around the world [1, 2]. While the use of Machine Learning (ML) algorithms in stratified medicine has its prior footprint in the field, recently there has been an advancement towards the acknowledgment of the need to have healthcare diagnosis systems using ML algorithms [3–5]. In the contemporary decade, the health industry has collected large amounts of medical data that can be analyzed through ML algorithms to appraise different patterns, to make intelligent diagnosis systems, and to identify important insights and adopt appropriate solutions [6].

Amidst all the other diseases, cardio-vascular diseases (CVDs) are the leading cause of death globally. According to the World Health Organization (WHO) an estimated 17.9 million people died from CVDs in 2019, representing 32% of all global deaths. Of these deaths, 85% were due to heart attack and stroke [7]. CVD is a general term for conditions affecting the heart or blood vessels which are usually associated with a build-up of fatty deposits inside the arteries (atherosclerosis) and an increased risk of blood clots.

Nevertheless, in today’s busy and fast-moving world, most people usually do not get a medical check-up unless they face any major health-concerning issues. Similarly, most of them do not have regular heart check-ups because the manual ways to get these check-ups done are both time-consuming and inconvenient. The aftermath of not knowing their present heart condition causes severe health conditions and, in the worst case sudden deaths. An intelligent system is thus required to keep track of one’s heart condition both conveniently and efficiently. Nowadays, the Internet of Things (IoT) has become an indispensable benefactor to the healthcare sector with its key features such as connectivity, sensing, reliability, linearity, and intelligence [8]. It is also a way to restructure modern health care by providing more individualized and preemptive care, sensing appliances, monitoring on tracking key health indicators such as pulse rate, blood pressure, and electrocardiogram (ECG) [9–11]. Several health-monitoring wearable sensors can be used for collecting real-time data from the human body and after further manipulation, these data can be converted into health records, which could be further used for CVD diagnosis, treatment, and postoperative remediation. Again, ML contrarily is an application of artificial intelligence (AI) that can apply what has been learned in the past to new data using labeled examples to predict future events [12–14]. Starting from the analysis of a known training dataset, the learning algorithm produces an inferred function to make predictions about the output values. It has various applications in healthcare, as it can be helpful in heart disease prediction at a primary stage so that proper precautions can be taken on time [15–17]. A person can know the risk of having CVDs through the use of ML by analyzing the medical records extracted from their body using wearable IoT devices. Despite all these technological progressions in healthcare systems, the application of AI and IoT is yet to be combined to create a friendly user end system that automates manual heart check-ups and gives predictions of having CVD in the future.

Again, Most of the prior research in this area mainly focused on developing better-performing ML algorithms only, whereas no embedded hardware systems were developed for acquiring real-time data from the users and testing the algorithm’s performance on the data collected from the users. In most of the previous research, no proper user-friendly interface was developed to give the user of the system ease and control over the hardware system and to show the results on the user end. A complete system that gives users control of the system and lets them check their heart conditions by themselves was mentioned nowhere. Additionally, the previous works were only done on the detection of CVD, classifying the users in two classes of having and not having the disease. To bridge this gap, the evolution of such a system is required that can:

- Efficiently extracts crucial heart condition measuring indicators (e.g., Pulse Rate, Blood pressure, ECG) from the human body in real-time.
- Process the real-time data in cloud storage and transform it into medical records.
- Feed those medical records to an ML model which can predict the risk of a person having CVD in the future.
- Exhibit the end-users the risk zone (level) classification and health-monitoring updates by an application.

Therefore, the objectives of this research are: Firstly, to deduce the best performing ML technique for predicting the risks of having CVD. Secondly, to develop a system consisting of a wearable device and a mobile application for CVD risk level prediction. Finally, to evaluate the functional and usability performance of the application in terms of effectiveness, efficiency, and user satisfaction. In other words, the research will investigate the answers to the following questions: (a) How to deduce a best-performing ML algorithm that can predict the risk of cardio-vascular diseases? and (b) What kind of system (Mobile IoT Application) may effectively predict the CVD risk level with a higher yield of usability and user satisfaction?

To attain the objectives as well as achieve effective answers to the stated research questions, ten ML algorithms were explored and an ensemble model with the best-performed ones was selected. Then, a wearable system was developed to predict the risk of having CVD for the real-time data acquired. Finally, the system was evaluated by physicians and patients to assess its effectiveness, efficiency, and satisfaction.

As such, the contributions of this research can be highlighted as follows. Firstly, this research focuses on developing a wearable hardware device that can efficiently pull out crucial heart condition measuring indicators such as Pulse Rate, Blood pressure, and ECG data from the user’s body in real-time. Secondly, a mobile application has been developed that can not only control the wearable hardware device and receive data from it in real time but also process the real-time data in cloud storage and transform it into medical records. Thirdly, the best-performing ML technique is deduced among several classifying algorithms for predicting the risks of having CVD. Finally, the functional and usability performance of the application has been evaluated in terms of effectiveness, efficiency, and user satisfaction.

## Related works

A number of studies were conducted on heart disease prediction with neural networks and conventional ML techniques. For example, a study conducted by Dinesh et al. [18] on the prediction of CVD using ML algorithms analyzed four heart disease datasets of UCI and suggested Logistic Regression (86.5%). Similarly, a study using hybrid ML techniques for heart disease prediction implemented by Mohan et al. [19] had suggested a hybrid-random forest with a linear model (88.7%) on the UCI Cleaveland dataset. In another study, Gavhane et al. [20] proposed a model-built UCI dataset stating Random Forest (89%) as the best predictor of heart disease, while Pandi-Jain et al. [21] showed an accuracy of about 100% to predict heart disease could be reached by Multilayer Perceptron Neural Network with backpropagation using 40% of UCI Cleaveland dataset as training data. Krittanawong et al. [22] carried out a meta-analysis to evaluate and describe the overall prediction capacity of Machine Learning algorithms in cardio-vascular diseases (coronary artery disease, heart failure, stroke, and cardiac arrhythmia). As an outcome, they found that the deep learning model, as well as boosting models and SVM models for predicting CAD and stroke risk, is promising.

Few research studies on disease prediction systems have proposed IoT-based solutions for ML analysis of various real-time sensor data. For example, Khan [23] proposed an IoT framework for heart disease prediction adopting a Modified Deep Convolutional Neural Network (MDCNN). It was an IoT-enabled wearable heart disease prediction system that classified the sensor data into two categories (Normal, Abnormal) and notified the concerned doctor in case of any abnormalities. Similarly, Ganesan and Sivakumar [9] proposed an IoT-based heart disease prediction and diagnosis model where data gathered from IoT devices are stored in cloud and then analyzed through ML algorithms for heart disease diagnosis. In another study, Ani et al. [24] proposed an IoT-based patient monitoring system for stroke-affected people to minimize future recurrence of the disease by alarming the doctor on variation in risk factors of stroke disease, while Mishra et al. [10] proposed a heterogeneous IoT body area network and communicated ECG and heart bps to the server for feeding them into prediction models built with random forest classifiers on the UCI dataset. IoT was also incorporated with an adaptive neuro-fuzzy inference system by Mohammad Ayoub and Fahad [25] to diagnose heart disease with an accuracy of 99.45%.

In addition to IoT-based solutions, a study by Ali et al. [26] suggested ontology-based recommendations to patients upon their clinical records and collected real-time data. Again, a moderate number of studies have suggested heart disease diagnosis solely from ECG data. For example, Thai et al. [27] proposed a heart disease diagnosis system based on a large dataset with an AD8232 ECG sensor, capable of removing noises from raw ECG signals and extracting vital features for performing diagnosis for supporting patients and physicians. Similarly, Kamaruddin et al. [28] showed CVD detection through SVM and Neural Network by analyzing features extracted from processed ECG signals. Furthermore, deep learning methods were also proposed by Yu-Sheng et al. [29] along with IoMT (Internet of Medical Things) to develop a valvular heart disease screening system using the relation between human blood circulation and body surface temperature. But the process required assessing medical test reports by a professional doctor or licensed examiner.

In some of the relevant studies, the usage of user-end systems is mentioned consisting of a hardware segment that can get integrated with a software segment. Nashif et al. [30] conducted a study on heart disease detection using ML algorithms and proposed a real-time cardiovascular health monitoring system (an application UI) at both doctor and patient ends for information collection as user input in addition to wearable sensors. Also, the paper had suggested some extraneous sensors having less contribution in the disease prediction model, making the wearable device immobile.

Based on these studies, several gaps are perceived. First, a prodigious amount of ML classifier usage to develop a prediction model for CVD prediction, while very few studies adopted different Artificial Neural Network concepts. Although the earlier systems were not concerned about real-time data for prediction purposes, few recent studies have proposed real-time decision-making using IoT technology, while a handful of studies have considered the IoT or sensor technology in the most beneficial way to make the hardware device wearable and mobile.

Second, almost all the prediction models were trained and tested with the UCI Cleveland dataset for heart disease, possessing fewer data points than the entire dataset. Using a relatively small test and training dataset provided a satisfactory accuracy that could be the result of overfitting, which is an excessive adjustment to the training data or outliers where few data points are noticeably different from the rest.

Third, few studies focused on proposing systems using the prediction models to be used by the doctors and primarily by the patients as the end-users. Nevertheless, none of those proposed systems had an adequate user end framework that could benefit the users by showing them prediction results and health monitoring features.

Fourth, most of the studies have focused on proposing an ML model mainly to predict CVD i.e. classify users into two levels, representing the presence or absence of CVD rather than classifying the risk level of having disease into multiple levels so that precautionary measures can be taken beforehand.

Thus this research focuses on developing an IoT and ML-based system for predicting the risk level of having cardio-vascular diseases and also evaluating the system with physicians and patients feedback to validate in terms of effectiveness, efficiency and satisfaction.

## Research methodology

**Fig. 1:**
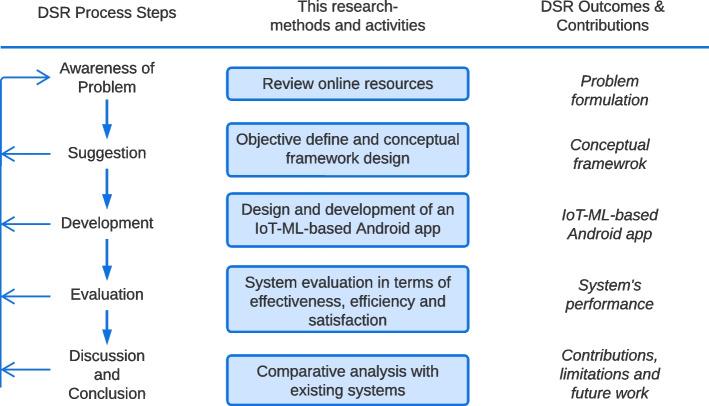
Overview of the research

To attain the objectives of this research, a Design Science Research (DSR) [31] approach has been incorporated. This process diverges into five steps: (1) awareness of the problem, (2) suggestion, (3) development, (4) evaluation and (5) discussion and conclusion, as shown in Fig. 1. The methodology followed in our research is classified into five methods and activities, linked with the corresponding steps of the DSR approach, along with the outcomes of this research (last column).

In the first step, awareness has been gathered based on the current situation of the problem, that is, the measurement of the CVD risk level. For this purpose, we have reviewed related research articles, trailed existing mobile applications and perceived the research gap. To bridge this gap, the objectives of this research are then formulated. This step is briefly discussed in the Related works section of this article.

The next step, suggestion, a conceptual framework has been proposed to achieve the objectives of this research. Three modules have been integrated for this purpose. Further discussion on the Conceptual Framework has been illuminated under System development section.

In the third step, a prototype of our system has been developed to achieve the objectives mentioned in the earlier steps. We developed three modules as stated in the Conceptual Framework section in this step. A Data Acquisition model is introduced to collect real-time data from the user, based on which a Predictive Analysis module can calculate the risk level of the user’s risk of having CVD. A User Interaction module is also developed in this step to integrate all these modules and present that in a user-friendly way. All these modules are discussed under the System development section.

Later on, the developed system was evaluated in terms of Efficiency, effectiveness and satisfaction in the fourth step of our methodology. The evaluation study was replicated with 40 participants and both the qualitative and quantitative data were collected and analyzed. A detailed description of the evaluation process is discussed in the System evaluation section of this article. And finally, in the fifth step, the outcome of our research is stated along with the comparison with existing systems in the Discussion Section of this article. To conclude the study, a brief overview of the reflection of design, development and evaluation is included along with the contributions and limitations of this study in the Conclusion section of this article.

## System development

**Fig. 2:**
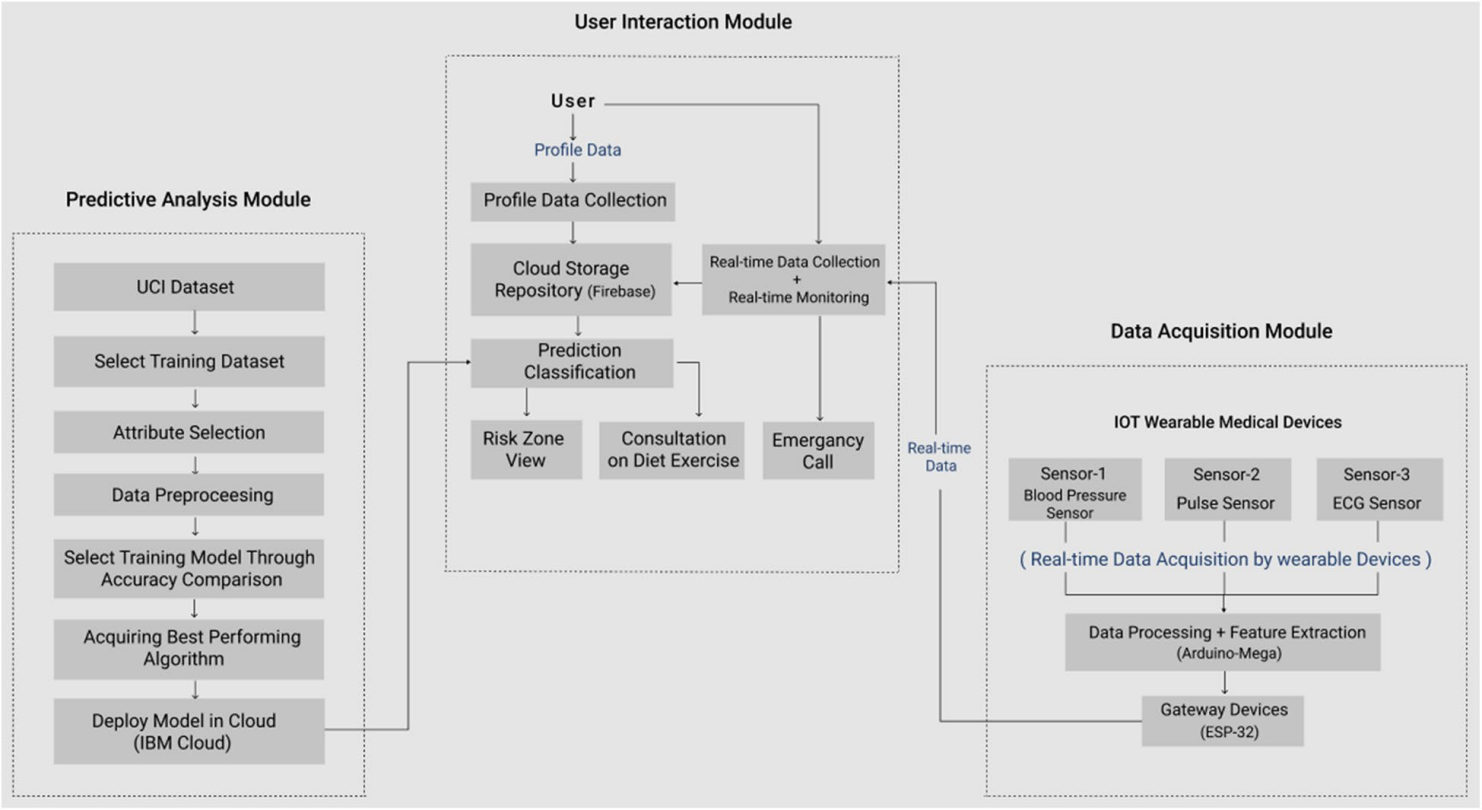
Conceptual framework of the proposed system ‘Predictis’

To develop an IoT and ML-based application for CVD risk level prediction system, a conceptual framework is proposed and shown in Fig. 2.

The system is initially divided into three modules. In the *Predictive Analysis Module* the best-performing ML algorithms will be ensembled through accuracy comparison among several ML algorithms. *Data Acquisition Module* comprises a wearable device consisting of three bio-medical sensors: a blood pressure sensor for measuring the blood pressure, a pulse sensor for getting the pulse rate, and an ECG sensor to measure the heart’s electrical activity in real-time. A mobile application will be developed in the *User Interaction Module* that allows the user to collaborate with the whole system. It will connect the *Data acquisition Module* with the *Predictive Analysis Module* and will enable the user to observe their real-time data and see the prediction result along with the consultation. The modules are discussed elaborately in the following sections.

### Predictive analysis module

For developing the system, at first the best performing prediction algorithms are selected by evaluating ML models built on open source heart disease data (UCI dataset). The purpose of this module is to build suitable prediction models for two types of classification task: a) two-zone classification indicating presence (denoted by red zone) or absence (denoted by green zone) of CVD, b) three-zone classification indicating low (green zone), moderate (yellow zone) or high (red zone) risk of CVD. The ML models were built in the following stages: Data Collection, Data Preprocessing, Developing and Evaluating the Prediction Models. The phases are briefly discussed in the following subsections:

*A. Data Collection:* This study used the dataset which contains four databases concerning heart disease prediction prepared by Cleveland Clinic Foundation, Hungarian Institute of Cardiology (Budapest), V.A. Medical Center (Long Beach, CA) and University Hospital, Zurich (Switzerland) [32]. Each database has the same instance format and the dataset has 920 samples in total. The “num” field of the dataset refers to the presence of heart disease in the patient. In the original dataset, it is integer-valued from 0 (no presence) to 4. For classification in three levels, level 0 of the ‘num’ field has been considered as green zone, 1 as yellow zone and level 2 to 4 as red zone. From 920 samples of the dataset, there are 413 green zone values, 258 yellow zone values, and 249 red zone values. For classification in two zones, level 0 of the ‘num’ field has been considered as green zone and level 1 to 4 as red zone having 413 and 509 samples respectively.

Thus, among the 14 attributes of the dataset, 12 of them have been used for the developed system. The description of all 14 features is provided in Table 1. As one of the goals of this study is to create a system that can forecast CVD risk levels without requiring a visit to the hospital or any medical investigation, attributes ‘ca’ and ‘thal’ have been omitted; because these features require users to do medical tests like fluoroscopy and thallium stress test.

**Table 1:** Description of features of the dataset

Feature	Attribute name	Domain	Data type	Mean	STD	Missing Values (%)
Age	age	Age in years :29-77	Real	54	9	0.00
Sex	sex	Male=1 ,Female=0	Binary			0.00
Chest pain type	cp	1=typical angina	Nominal			0.00
		2=atypical angina				
		3=non-anginal pain				
		4=asymptomatic				
Resting Blood Pressure in mm/Hg	trestbps	94-200	Real	131.344	17.862	6.41
Serum Cholesterol in mg/dl	chol	126-564	Real	249.659	51.686	3.26
Fasting blood sugar > 120 mg/dl	fbs	1=yes,0=no	Binary			9.78
Resting ECG observations	restecg	0=normal	Nominal			0.22
		1=having ST-T wave abnormality (T wave inversions and/or ST elevation or depression of > 0.05 mV)				
		2=showing probable or definite left ventricular hypertrophy by Estes’ criteria				
Maximum heart rate achieved	thalach	71-202	Real	149.678	23.166	5.98
Exercise-induced angina	exang	1=yes,0=no	Binary			5.98
ST depression induced by angina relative to rest	oldpeak	0-6.2	Real	1.05	1.145	6.74
Slope of the peak exercise ST segment	slope	1=upsloping	Ordered			33.58
		2= flat				
		3=downsloping				
Number of major vessels colored by fluoroscopy	ca	Number of vessels: 0,1,2,3	Real			66.43
Thallium stress test result	thal	3 = normal; 6 = fixed defect;	Nominal			52.83
		7 = reversible defect				

*B. Data Preprocessing:* The data preprocessing was carried out in two phases. In the first phase missing values problem was handled; while in the second phase rescaling of both the numerical and categorical values was done. The phases are described below.

**Fig. 3:**
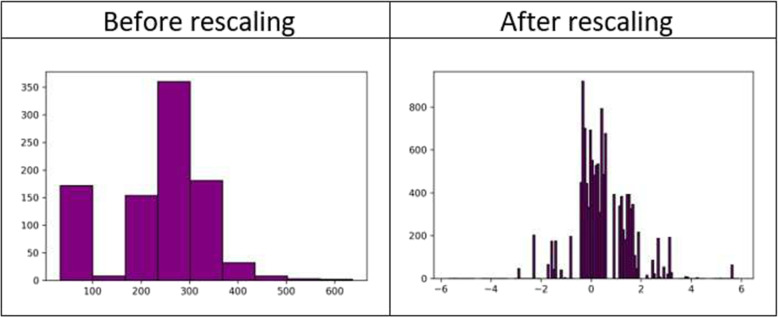
Histogram plots of the feature ’Cholesterol’ of dataset before and after rescaling

- **Handling Missing Values:** The dataset contains structured data with many samples with null values which are demonstrated in Table 1 as percentages of missing values per attribute. Since the data are missing at random, the missing value problem has been handled using the Multiple Imputation by Chained Equations (MICE) method [33]. In this method, the dataset’s missing data is “filled in” (imputed) by the technique using an iterative cycle of predictive models. Each specified attribute with a missing value in the dataset is imputed using the other attributes in the dataset at each iteration. These iterations are done until it seems like convergence has been reached.
- **Rescaling the Data:** After the missing value imputation, the continuous features of the dataset have been rescaled to remove the outliers. The median (50th percentile), 25th percentile, and 75th percentile are all calculated to achieve this. The values of each variable are then reduced by the interquartile range (IQR), which is the difference between the 75th and 25th percentiles, and their median is subtracted [34]. This method of rescaling is known as robust scaling of data. The impact of this rescaling can be observed in Fig. 3 which shows the histogram plots of the feature ’cholesterol’ of the dataset before and after rescaling. The figure presenting the histogram of ’cholesterol’ feature before rescaling contains significant outliers while the histogram after rescaling has 0 as both mean and median and 1 as the standard deviation, resisting the pull of outliers. The categorical values of the dataset, are replaced with a numeric value between 0 and the number of classes minus 1. After the preprocessing of the dataset, it has been oversampled to balance the class distributions and split in a 70/30 train test split to develop the machine learning model.

*C. Developing and Evaluating the Prediction Models:* In this study, the prediction models are generated using the training dataset whereas the performance of prediction models is evaluated for the unknown dataset (test set).

Through meticulous analysis, an ensemble-based ML model for predicting CVD is developed in two phases. In the first phase, several ML algorithms are analyzed and five best-performed algorithms are selected and in the second phase, the stacking classifier is built with the selected algorithms. The phases are discussed in the following subsections:

*a. Selecting best performing ML algorithms:* For selecting the best performing algorithms, ten ML algorithms are explored and analyzed using the dataset. All classifiers have been used after proper hyperparameter tuning in the Grid Search cross-validation method on the training dataset. Nonetheless, the performance of each of the models for the testing instances is measured in terms of precision, recall, and f1 score, and the results are shown in Table 2 for three and two-zone classification.

**Table 2:** Evaluation of models through precision, recall and F1 scores for two and three zone classification

Model	Two level-classification			Three level-classification		
	Precision	Recall	F1 score	Precision	Recall	F1 score
KNN	0.877	0.881	0.878	0.697	0.690	0.690
Naive Bayes	0.839	0.845	0.840	0.685	0.679	0.681
Random Forest	0.851	0.847	0.849	0.645	0.645	0.644
Support Vector Machine	0.855	0.855	0.855	0.749	0.745	0.742
Gradient Boosting	0.840	0.836	0.838	0.624	0.625	0.625
SGD Classifier	0.837	0.792	0.800	0.564	0.588	0.563
XGB Classifier	0.867	0.865	0.866	0.778	0.777	0.777
MLP classifier	0.861	0.860	0.861	0.739	0.731	0.726
Decision Tree	0.838	0.840	0.839	0.571	0.553	0.554
AdaBoost	0.855	0.859	0.856	0.660	0.662	0.661

From the results depicted in Table 1, it is evident that KNN, XGB, MLPC, AdaBoost, and SVM performed considerably better (F1 score 85%+) for two-level classification and XGB, SVM, MLPC, KNN, and Naïve Bayes performed considerably better (F1 score 66%+) in three-level classification. Thus five algorithms for both two and three-level classification are selected to build the ensemble stacking model in the next phase.

*b. Developing the stacking classifier:* To create an efficient ensemble model both two and three zone-classification models are developed with the Stacking method of ensemble learning integrating the best-performing algorithms. In both cases, the Logistic Regression algorithm is used as a meta-classifier in the Stacking algorithm. To find the best performing combination of the base-learners several models with three to five stacked base-learners are created with the resultant algorithms of phase one. The results of performance evaluation for test data measured in precision, recall and F1 scores are provided in Tables 3 and 4 for two and three zones respectively. The combinations with repeated values of considered evaluation metrics have been excused in the resultant tables.

**Table 3:** Performance evaluation for stacking classifiers (two-level classification)

Stacking classifiers	Precision	Recall	F1 score
KNN, XGB, ADA	0.888	0.890	0.889
KNN, XBG, SVM	0.878	0.878	0.878
KNN, XGB, MLPC	0.910	0.910	0.910
KNN, XGB, MLPC, ADA	0.871	0.875	0.873
XGB, MLPC, ADA, SVM	0.882	0.886	0.884
KNN, XGB, MLPC, ADA, SVM	0.872	0.873	0.872

**Table 4:** Performance evaluation for stacking classifiers (three-level classification)

Stacking classifiers	Precision	Recall	F1 score
SVM, MLPC, XGB	0.749	0.747	0.746
SVM, MLPC, KNN	0.713	0.713	0.712
SVM, KNN, XGB	0.816	0.793	0.804
MLPC, KNN, NB	0.734	0.733	0.731
MLPC, KNN, XGB	0.767	0.766	0.765
SVM, MLPC, KNN, NB	0.749	0.747	0.746
SVM, MLPC, KNN, XGB	0.798	0.797	0.797
MLPC, KNN, NB, XGB	0.781	0.782	0.781
SVM, MLPC, KNN, NB, XGB	0.798	0.797	0.797

From the resultant tables, it is perceivable that both two and three zone-classification models developed with the Stacking method of ensemble learning performs best in testing data with an F1 score of 91% and 80.4% respectively.

**Fig. 4:**
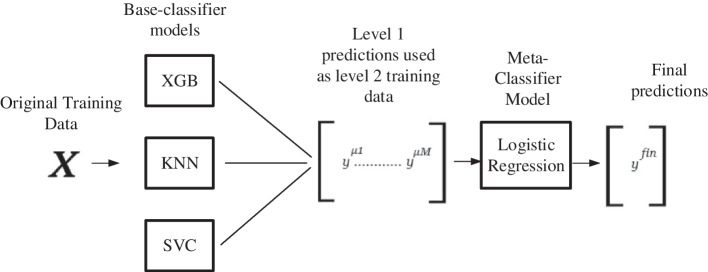
Architecture of proposed three-level stacking classifier model

**Fig. 5:**
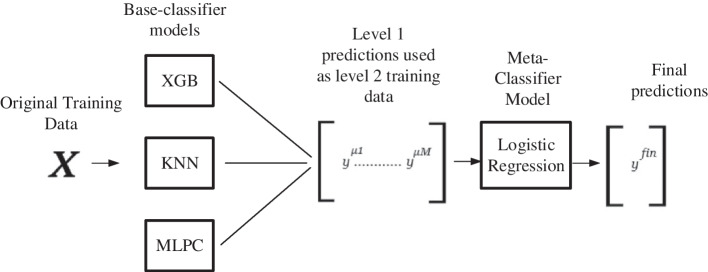
Architecture of proposed two-level stacking classifier model

The best performing stacking classifier from Tables 3 (F1 score 91%) and 4 (F1 score 80.4%) have been used as proposed prediction models *Predictive Analysis Module*. Thus, for the proposed two-zone stacking model XGB, KNN, and MLP Classifier are base-classifiers and for three-zone models, SVC has been used along with XGB and KNN as the same. The architecture of the proposed models for three and two-zone classification are provided in Figs. 4 and 5 respectively.

**Fig. 6:**
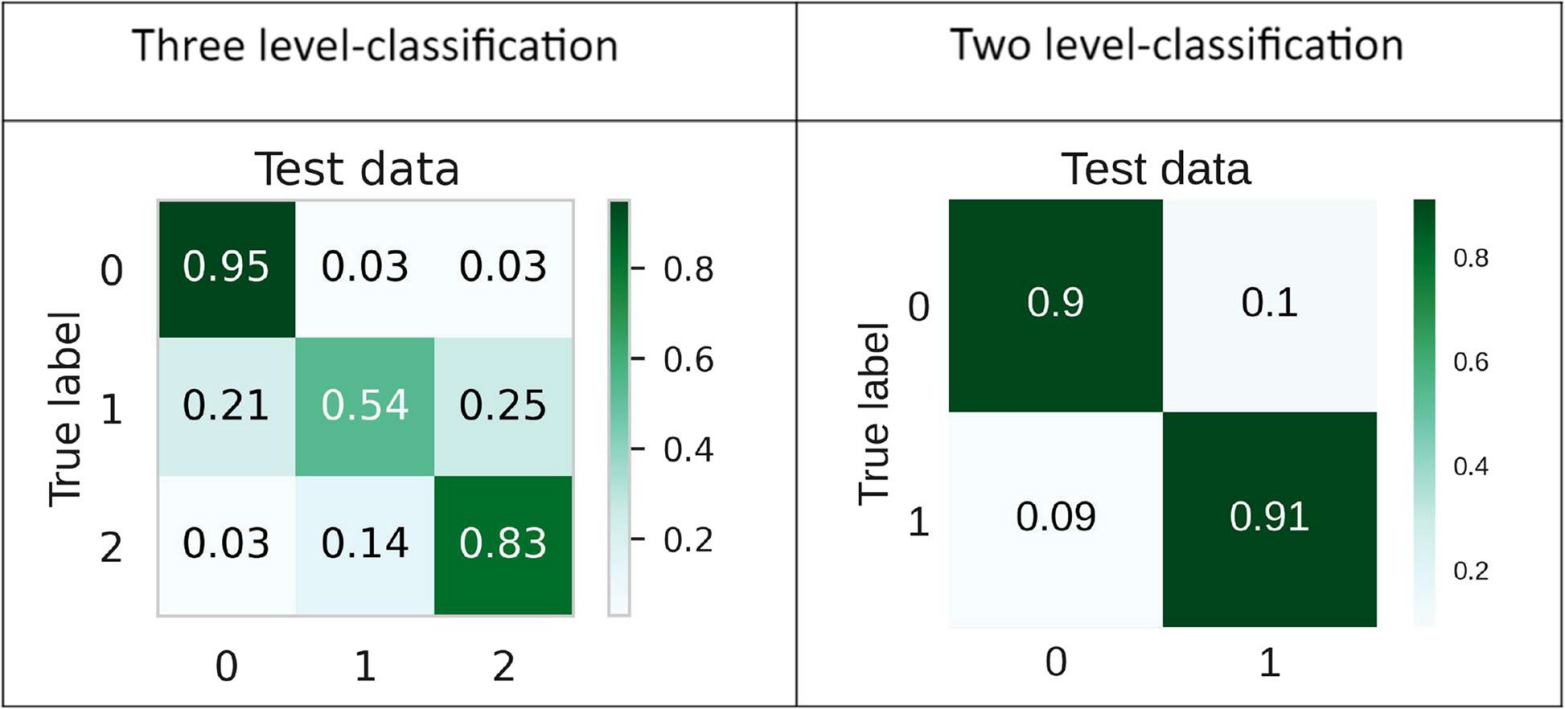
Performance of developed models through Confusion-Matrix for two and three-level classification using the proposed stacking classifier

**Fig. 7:**
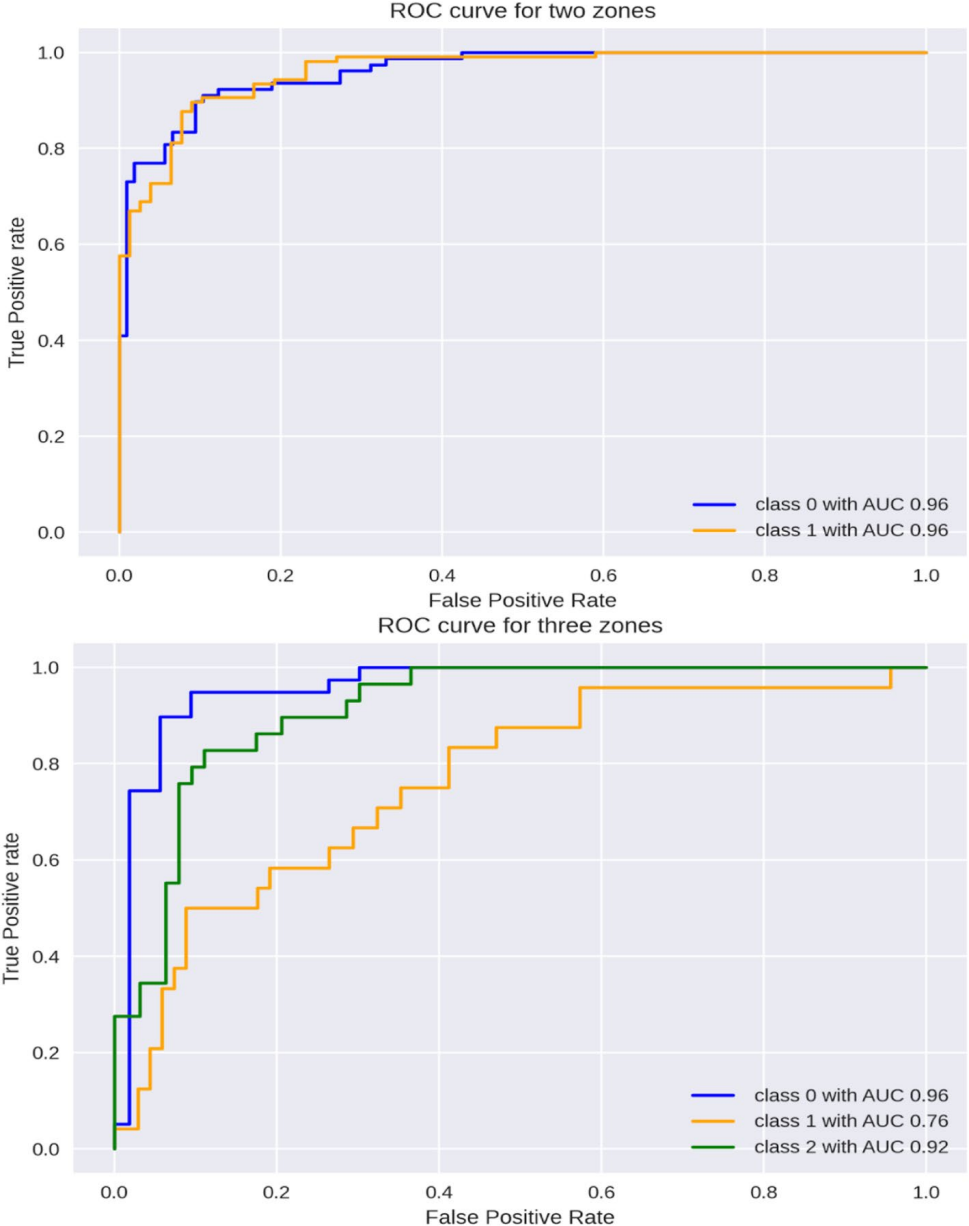
Performance of developed models through ROC-AUC for two and three-level classification using the proposed stacking classifier

The proposed models’ performance is also measured through the ROC-AUC score. The proposed three-zone classification model has an AUC score of 0.96 for green (0) zone, 0 .76 for yellow (1) zone and 0.92 for red (2) zone. The proposed two-zone classification model has an AUC score of 0.96 for both red (1) and green (2) zones. The results are shown in Figs. 6 and 7. Further evaluation of the proposed models’ performance by confusion matrices has been shown in Fig. 6. However, it can be concluded from several performance evaluation techniques that the proposed two-zone classification model performs better than the three-zone classification model. The proposed two-zone classification model serves as a presence-absence predictor of CVD and the proposed three-zone model as a risk-level predictor of CVD in the cloud for the developed system.

### Data acquisition module

**Fig. 8:**
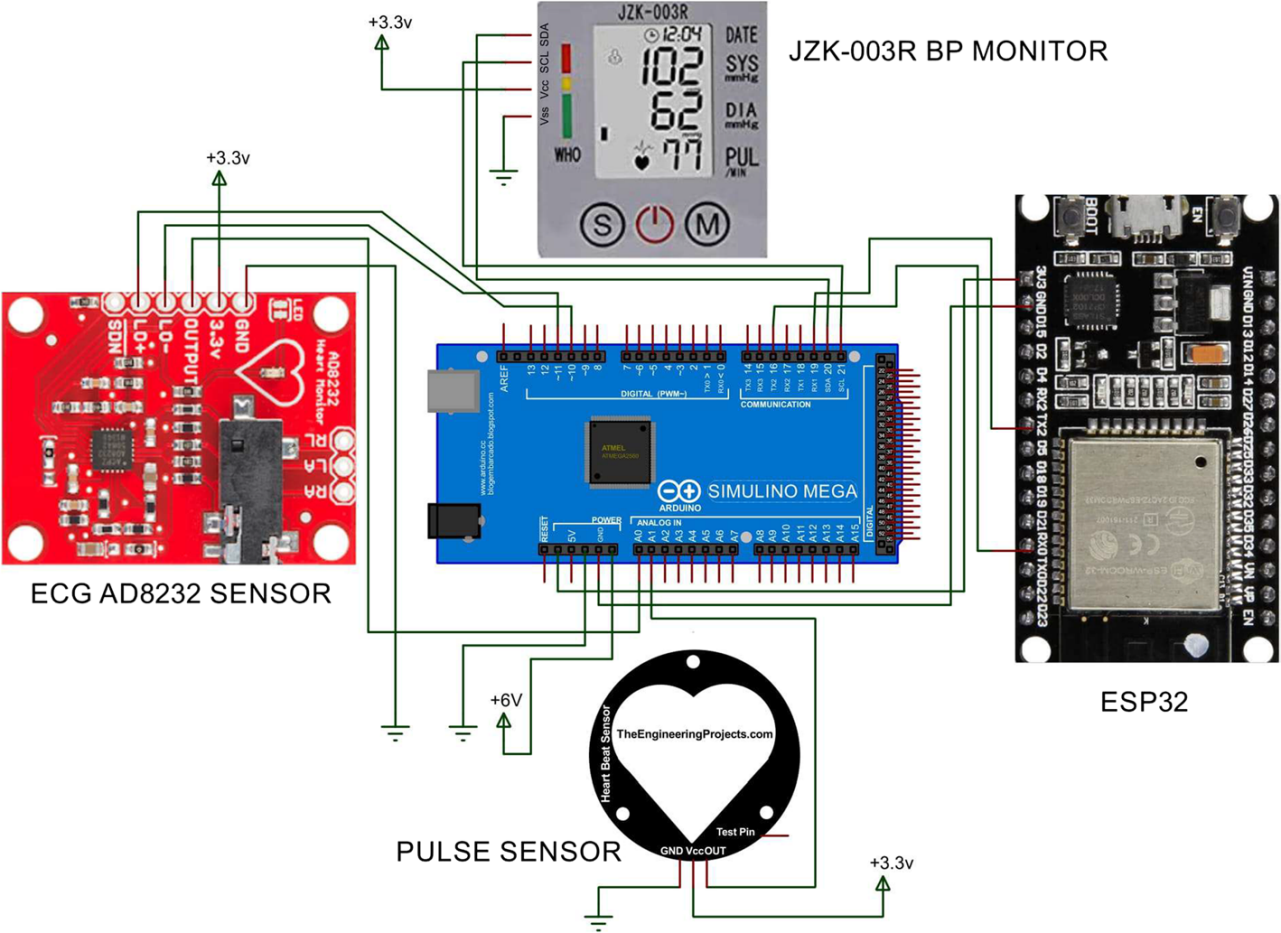
Circuit diagram of the Predictis wearable system

At the completion of the predictive analysis module, implementation of the data acquisition module started. In this module, a wearable health-monitoring system named ‘Predictis’ is developed. Figure 8 shows the circuit diagram of the Predictis wearable system. The system consists of an Arduino Mega 2560, an ESP32 Bluetooth and wi-fi module, and some biomedical sensors. In the Predictis, Arduino Mega is used as a microcontroller and ESP32 for Bluetooth communication. The pulse sensor is used for fetching the continuous pulse rate of the user. The ECG sensor is used for capturing small electrical signals from the heart. Then the ECG signal is processed to extract requisite features like Oldpeak, Slope, RestECG, etc. Again, for measuring a reliable blood pressure value, an automatic blood pressure monitor is used.

**Fig. 9:**
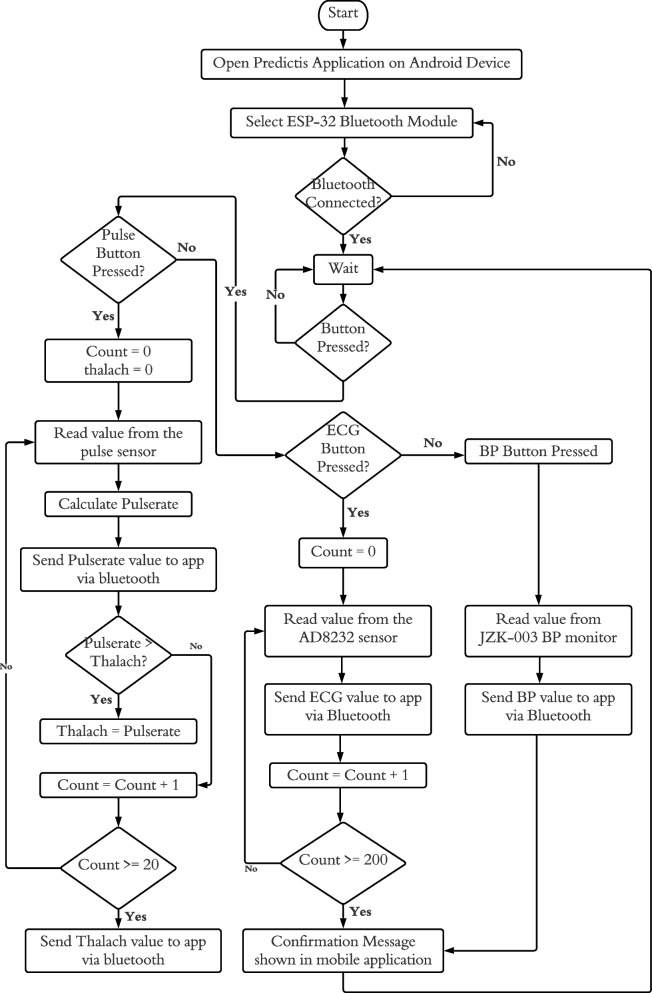
Flow diagram of the developed hardware system

The data collecting module is depicted in the flowchart illustrated in Fig. 9.

**Fig. 10:**
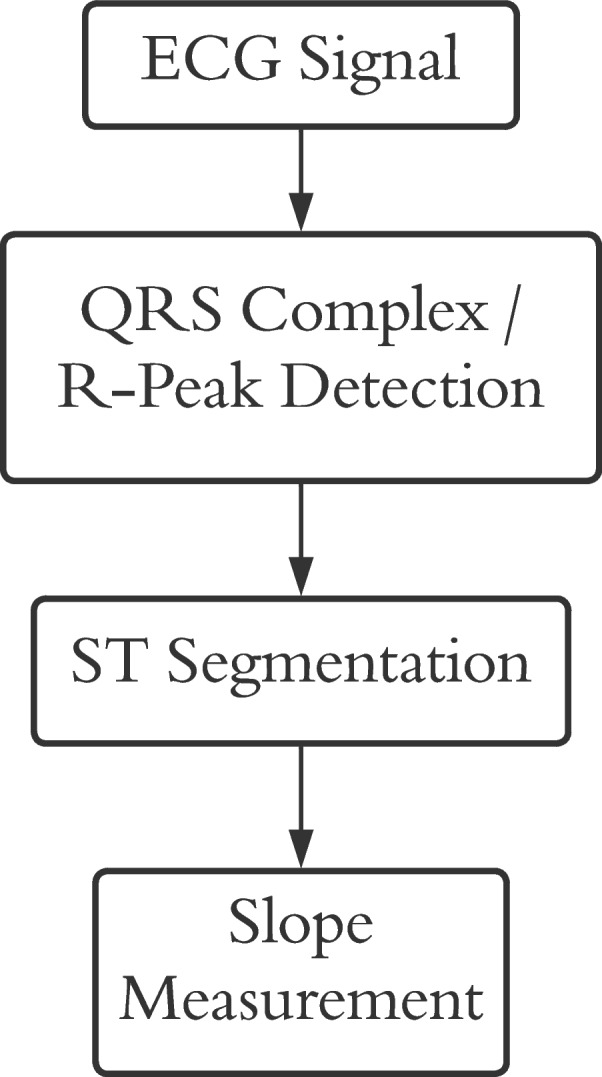
ST-segment Slope Measurement Procedure

The ECG signal is further processed using MATLAB installed in a VM in the cloud for extracting the value of the Slope of the ST-segment. The process of extracting the slope of the ST-segment is illustrated in Fig. 10.

To measure the slope of the ST segment, at first the QRS complex/R-peak needs to be detected. The Pan-Tompkins algorithm detects QRS complexes with great efficiency [35]. The algorithm consists of different phases. At first, the signal is passed through a bandpass filter consisting of high-pass and low-pass filters in cascade to increase the signal-to-noise ratio.

The equation of the low pass filters can be formulated as the following where the transfer function can be denoted as *H*(*z*) and the output function can be denoted as *y*(*n*):

1 $$\begin{aligned} H(z)=\frac{(1-z^{-6})^2}{(1-z^{-1})^2} \end{aligned}$$

2 $$\begin{aligned} y(n) = 2y(n-1) - y(n-2) + x(n) - 2x(n-6) + x(n-12) \end{aligned}$$

The equations of the high pass filters can be denoted as:

3 $$\begin{aligned} H(z)= \frac{1}{32} \frac{(1-z^{-32})}{(1-z^{-1})} \end{aligned}$$

4 $$\begin{aligned} y(n) = y(n-1) - \frac{1}{32} x(n) - x(n-16) - x(n-17)+ \frac{1}{32} x(n-32) \end{aligned}$$

After the noise cancellation is completed using cascading lowpass and highpass filters Then the signal is passed through a derivative filter which provides information about the slope of the QRS complex.

The derivator can be mathematically represented as:

5 $$\begin{aligned} H(z) = \frac{1}{10} (x+z^{-1}-z^{-3}-2z^{-4}) \end{aligned}$$

6 $$\begin{aligned} y(n) = \frac{1}{8}[2x(n)-x(n-1)-x(n-3)+2x(n-4)] \end{aligned}$$

After that the filtered signal is squared to magnify the dominant R-peaks and reduce the possibility of any garbage data. Then a window of 150ms is chosen for sliding window integration to extract the QRS complex.

**Fig. 11:**
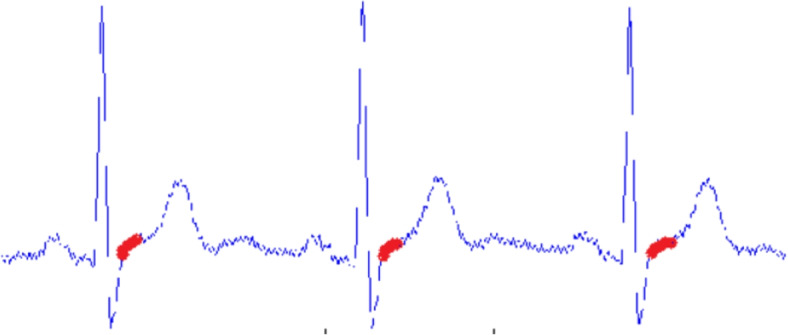
Extracted ST segment

After the QRS complex/R-peaks are detected, forging ahead towards ST segmentation is possible [35]. The time period of ST segments is generally 120ms [36]. As the ECG signal used for the developed system was of 350 Hz, the length of the ST segment was roundabout 42 samples. It is also perceived experimentally that the ST segment is found 20 samples after the R-peak. So, from 20 samples after the R-peak the 42 samples are recorded for further processing. Figure 11 displays the extracted ST segments.

The algorithm for ST-segment detection is given in Algorithm 1.

**Figure:**
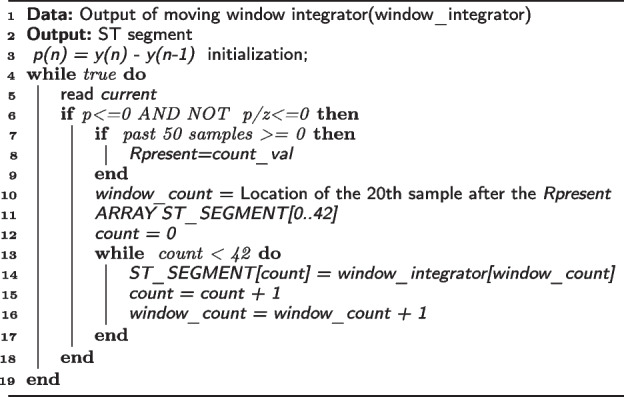
**Algorithm 1** ST SEGMENTATION PROCESS

After the segmentation is completed the slope of the ST segment is measured. For a particular ST segment, if X is last sample and Y is the first sample with total N number of samples then the slope will be:

7 $$\begin{aligned} tan \theta = \frac{X-Y}{N} \end{aligned}$$

**Fig. 12:**
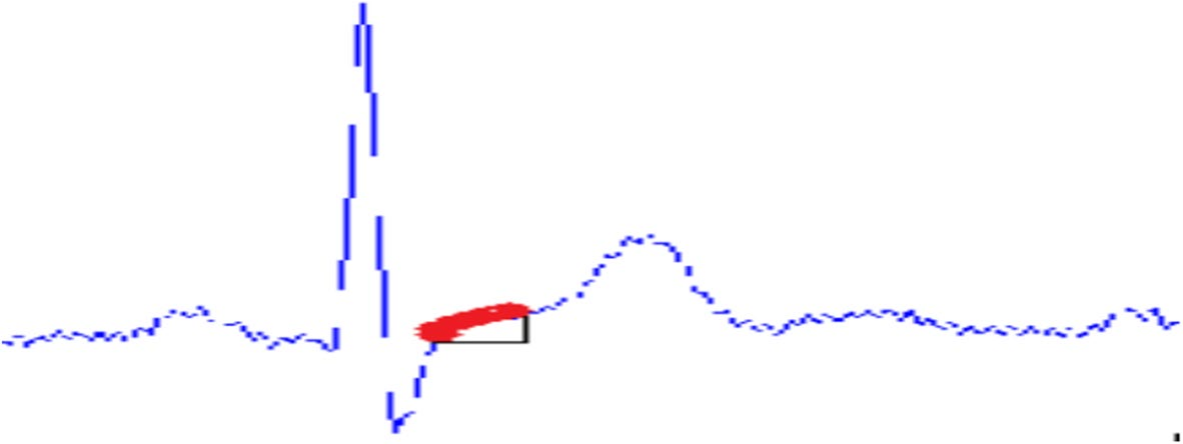
Slope of ST segment

As shown in Fig. 12 the highlighted part in red which is the slope of the ST segment is measured.

The old peak attribute of the dataset is the ST depression induced by angina relative to rest with values ranging from 0-6.20. The old peak feature has been predicted from the features it is dependent on by regression analysis for estimating the heart disease risk level. The correlation coefficient of the old peak with other attributes has been analyzed to select the features of the dataset it is dependent on. After that, a model built on the Support Vector Regression algorithm has been used to measure the value of old peaks from selected features.

On the other hand, the resting ECG observation is a categorical feature with values 0,1 or 2. The feature value has been measured from the Support Vector Classifier model upon proper feature selection from the dataset to find its dependency on other attributes.

Here, these two value prediction tasks have been treated as missing value imputation and the majority of the characteristic variables from which these two variables are predicted are categorical. As a result, we don’t anticipate extremely non-linear relationships. Only one variable is missing in both scenarios, making the SVM application relatively straightforward. Both the SVC and SVR algorithms are non-linear adaptations of linear techniques known as ’semi-parametric’ approaches because they have the efficiency of parametric techniques but the capacity to learn non-linear correlations, exactly like non-parametric methods [37]. Furthermore, the SVR recognizes non-linearity in the data and gives an effective prediction model in the case of old peak prediction [38].

**Fig. 13:**
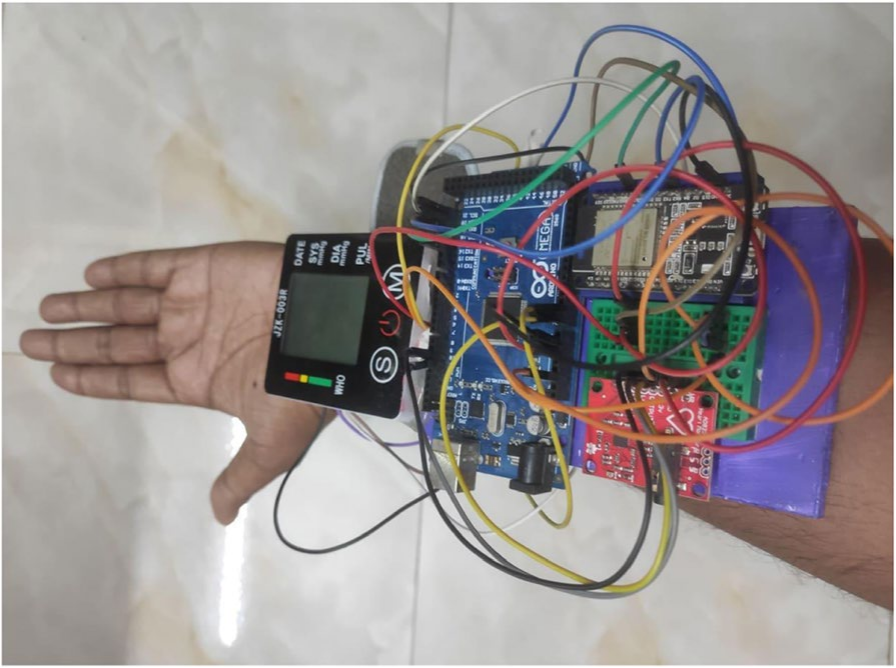
Developed Hardware System

The developed hardware device consisting of Arduino Mega, ESP32 and biomedical sensors are shown in Fig. 13.

Connecting the Android application to the wearable system is the first step in the hardware module. After connecting the wearable system to the Android app, it will remain in the wait mode until a button is pressed on the mobile application (see in Fig. 15b and c in user interaction module). The data collection operation will be finished by collecting the dataset properties PulseRate, Resting BP, Slope, Oldpeak, and RestECG in the correct order.

When the connect button on the Pulse Monitoring Device is pressed in the application, it calculates 20 consecutive pulse rates. The highest pulse rate out of them is known as Thalach. The Thalach and pulse rates are then transferred to the application through Bluetooth. When the connect button on the ECG Device is pressed in the application, 200 consecutive ECG data from the AD8232 ECG sensor are transferred to the application. When the connect button on the Blood Pressure Device is pressed in the application, the blood pressure (BP) is measured with the JZK-003 wrist blood pressure monitor and transferred to the application over Bluetooth.

### User interaction module

**Fig. 14:**
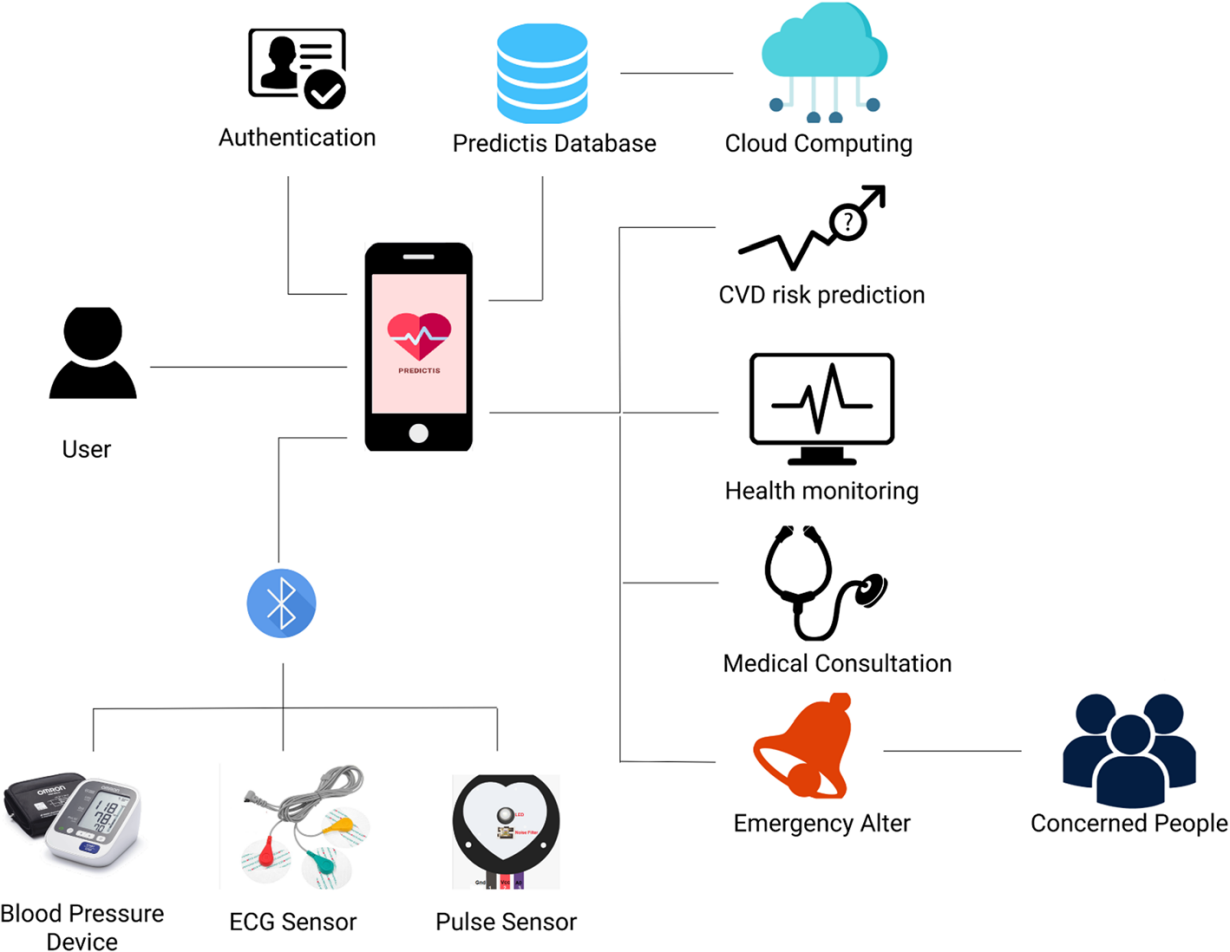
System architecture of the ‘Predictis’ system

An android mobile application has been developed for the end-users, incorporating the best performing ML model from the Predictive Analysis Module. Based on the prototype designed in Figma, a collaborative browser-based interface design tool, the application has been developed using Android Studio. The UI module facilitates the users to upload their profile and real-time data in the Predictis Database. The best performing ML model, deployed in IBM cloud, then formulate the prediction result based on these data collected from the user. The interaction module also enables the users to check their health condition in real-time and initiate emergency alerts if required. Consultations accumulated from doctors of this field are also provided to the users established on their CVD risk level. The System Architecture of this proposed system is shown in Fig. 14.

After a proper authentication process, users get associated with the features provided by the application.

**Fig. 15:**
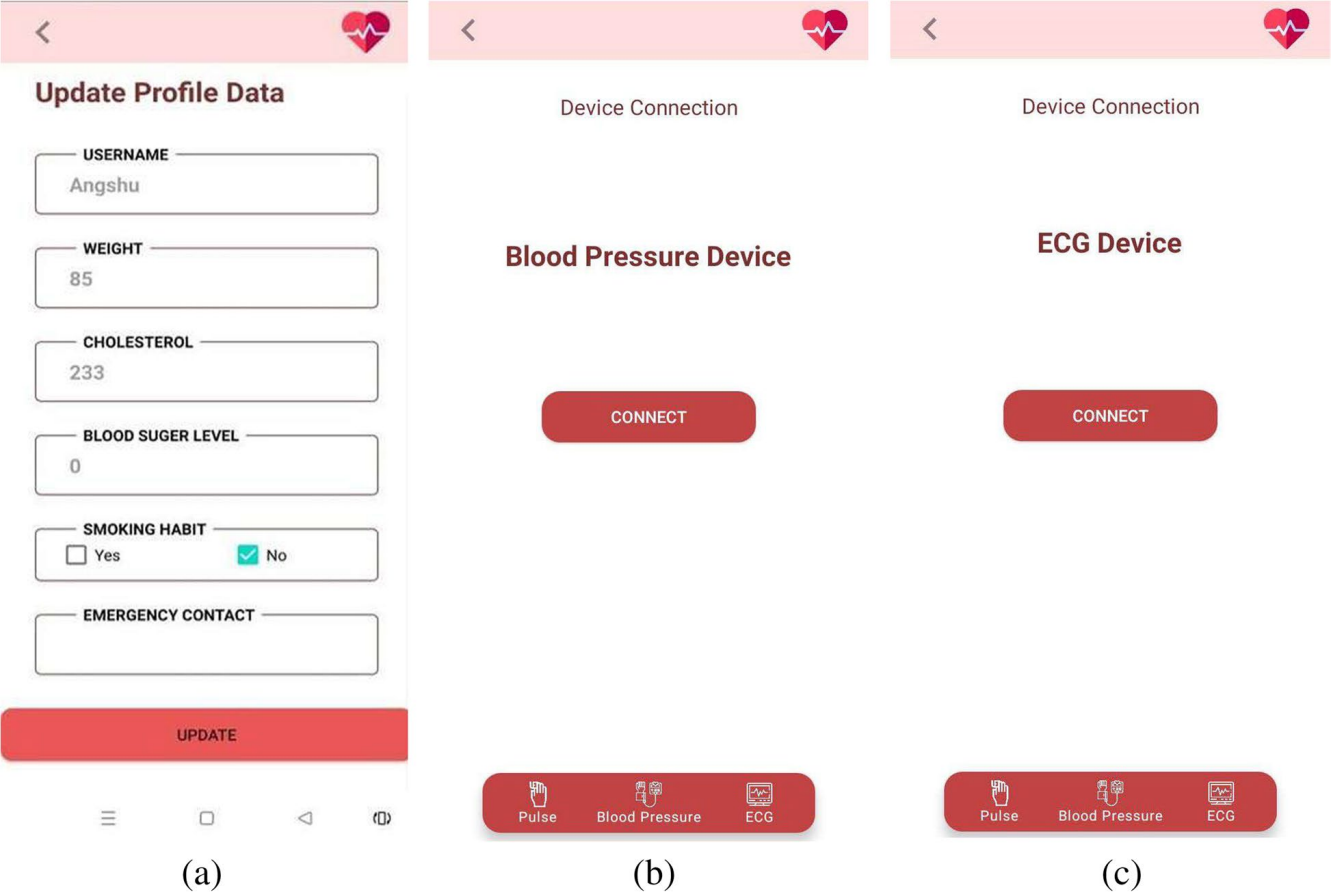
User interface for the device Connection

Upon the first login, some profile data including weight, cholesterol, fasting blood sugar, type of angina during exercise (if any) is collected from the users with an incorporated option to update them whenever required (see in Fig. 15a). This application enables the user to control hardware components using Bluetooth technology so that collection of data in real-time is possible. Users need to connect all three devices successively (see in Fig. 15b and c) and corresponding readings from the Data Acquisition Module will get collected and stored in the Firebase data repository.

**Fig. 16:**
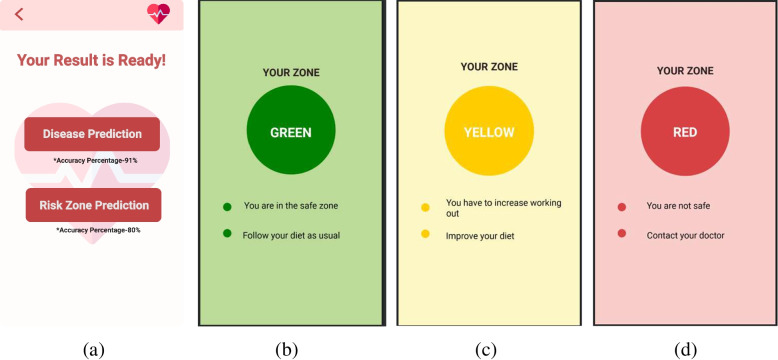
User interface for the risk Zone View

The ECG signal is further processed using MATLAB installed in virtual machine in cloud technology. Based on these data, users are able to see their possibility of having cardio-vascular diseases. Users can perceive the prediction result in two different categories based on their preferences (see Fig. 16a). For risk level classification, results are classified into green, yellow and red zones designating low risk to high risk of having CVD (see Fig. 16b, c and d). In case of two zone classification, users are categorized into red and green zones only representing the presence and absence of CVD.

**Fig. 17:**
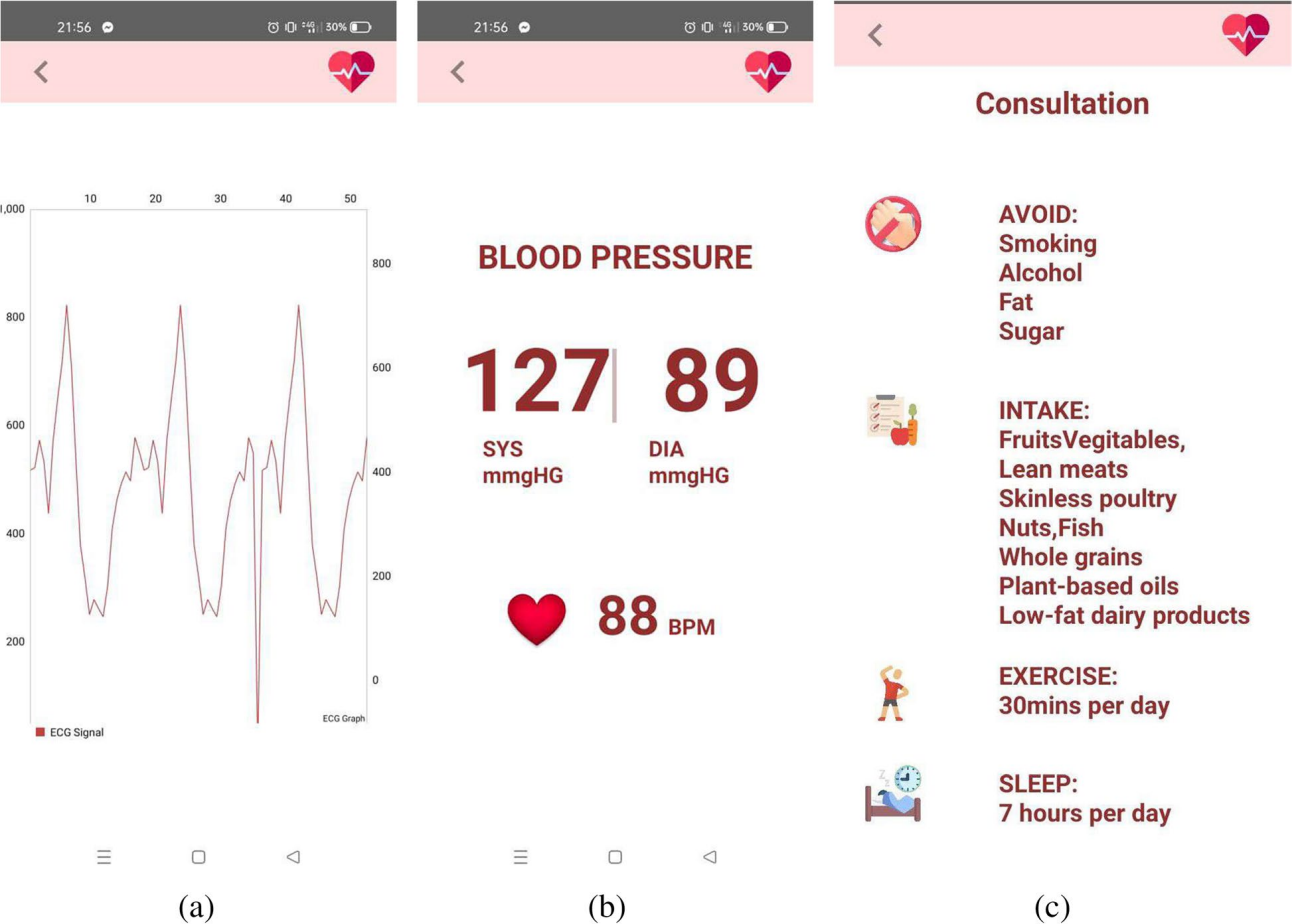
User interface for the monitoring and consultation

This framework also enables users to monitor their health conditions in real-time. Heart condition monitoring is possible using this application with ECG signals. There are also options for monitoring blood pressure, pulse and in case of any emergency corresponding alerts will be raised. Based on the health condition, users will also be provided with necessary consultation including diet plan, exercise routine etc. Monitoring and consultation UI of the application is represented in Fig. 17.

## System evaluation

An evaluation study was conducted to evaluate the performance and the usability of the developed system in terms of four indicators: I) Effectiveness, II) Efficiency, III) SatisfactionIV) Subjective feedback. To achieve the results of the evaluation first of all, a group of participants were gathered to test the system based on our designed study procedure. In the following subsections, profile of those participants and the procedure of the evaluation study are discussed in details.

### Participant’s profile

A total of 40 participants were invited to participate in this evaluation study. Among the participants 20 people were from the age group 50-70, 10 were from the age group 30-40 and 10 were from the age group 20-30. Among all the participants 19 were male and 21 were female. 14 of the participants were CVD patients and 13 were not aware of CVD. Almost 60% of the participants had an average experience of using mobile devices and computers for more than 4.06 years while 10% of the participants were novices in using mobile and computer.

### Study procedure

An evaluation process was organised in the Software Engineering Laboratory of the Authors’ Institute with the participation of the mentioned group of people. Individual sessions were arranged for each participant to evaluate the performance and the usability of the system. The subsequent steps were followed to conduct each session.

- Primitively, the participants were informed about the sole purpose of the evaluation procedure and also about the confidentiality of their data used in this evaluation study. Participants were encouraged to provide the correct information about their existing cardio-vascular diseases status. Information was collected through a survey form along with some biographical data.
- A live demonstration was shown concerning the features and usage of the *Predictis* device including the mobile application and the wearable device.
- Participants were provided with the Predictis device and asked to find out their risk level of having cardio-vascular diseases using the system. During the process, participants were allowed to ask for assistance from the research team. Each session was recorded as video, audio and screen record for additional evaluation purposes.
- To converge the honest point of view about the developed system, participants were motivated during the whole evaluation process. The evaluation study was concluded with a post-evaluation survey, conducted to accumulate user feedback in terms of performance, complexity, feasibility, consistency etc. In addition, System Usability Scale (SUS) [39] evaluation process was incorporated to gather user feedback so that the usability of the system can be outlined.

### Analysis and findings

In this section, the findings of the evaluation study is represented in terms of four indicators: I) Effectiveness, II) Efficiency, III) SatisfactionIV) Subjective feedback

#### I) Effectiveness

Profile and real-time data collected from the participants using Predictis device were used to evaluate the effectiveness of the system. The effectiveness has been evaluated with an association of 40 participants including 14 diagnosed CVD patients (positive) and 26 healthy people (negative). Among the 26 negative cases, 8 participants were found to have high cholesterol, blood pressure and elevated old-peak during exercise so they were determined to have a moderate risk of CVD. Table 5 presents the confusion matrix of the system concerning two-zone classification (green, red) as well as three-zone classification (green, yellow, red). The performance of the system, while risk level prediction, has been evaluated in terms of average accuracy, precision, recall and F1-score calculated from the confusion matrix. The system shows an accuracy of 87.5% (35 out of 40) among which accuracy for red-zone classification is 100% (14 out of 14). In addition, the responsiveness of the developed system is adequate, having a precision of 87.2% and recall of 91%. Finally, the F1 score of 87.7% conveys the effectiveness of CVD risk level prediction using real-life data samples.

**Table 5:** Confusion matrix for evaluation in effectiveness for three and two-level classification

True Labels /Predicted Labels	Three-level Classification			Two-level classification	
	Green	Red	Yellow	Green	Red
Green	13 [18]	0	5	23 [24]	1
Red	0	14[14]	0	3	13 [16]
Yellow	0	0	8 [8]		

**Fig. 18:**
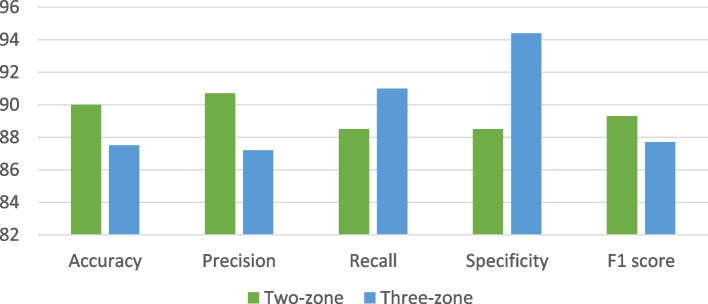
Effectiveness of the Prediction Model (three-level and two-level classification) of the developed application based on the participants’ dataset

For two-zone classification models, the same dataset has shown an accuracy of 90% (36 out of 40). The model can classify red-zone data 87% accurately (13 out of 16) and green-zone data 93% (23 out of 24) accurately. The precision is 90.7%, the recall is 88.5% and the F1 score is 89.3% which is comparatively better than the three-zone classification. Table 5 shows the results of the effectiveness of the system through confusion matrix and Fig. 18 shows the comparison of the effectiveness of the system’s two and three-zone classification by accuracy, precision, recall, specificity and F1 scores.

#### II) Efficiency

To check the quality and state of the system while predicting CVD risk levels, certain evaluation parameters are selected concerning the application efficiency, data acquisition module efficiency and overall system complexity.

- *Number of Clicks to complete the task:* To complete the whole prediction task, a total of 19 clicks were required. The evaluation result accomplished with the same participants’ group showed that minimum 19 clicks and maximum 22 clicks were made to complete the task with an average of 19.325 clicks. The minor deviation of 0.325 clicks between the optimal and the average number of clicks by the participants shows the efficiency of the mobile application.
- *Task Completion time:* The evaluation result concerning the efficiency of the system showed that participants took a minimum of 65.9 seconds and a maximum of 105.7 seconds to complete the prediction task with an average of 80.21 seconds. Almost two-thirds (25 out of 40) of the participants were able to complete the task within the average time, making the application functions easy to understand for all age groups.
- *Wearable device attachment time:* For real-time CVD risk prediction the participants were required to wear Predictis data acquisition module. The evaluation result showed that participants took 126.6 seconds on average to put on the sensors with a maximum time of 178 seconds and a minimum of 90.8 seconds, showing an indication of an efficient system.
- *Number of Attempts for completion:* The participants had taken 1.15 attempts on average to complete the whole task of connecting the wearable sensors and predict their CVD risk level while the optimal number of attempts should be one. 3 out of 40 participants (7.5%), especially participants with minor experience in operating mobile devices, took more than one attempt. Rest of the participants completed the whole task in an optimal number of attempts, showing the ease of use of the system.
- *Seeking help from the Researcher:* While predicting the CVD risk level from the application, 12.5% (5 out of 40) of participants asked for assistance and all of them were from the age group (50-70 years old). *“...Is my hardware collecting data properly?...”* was the most asked question while connecting to wearable devices. 15% (6 out of 40) of the participants asked for help while wearing the data acquisition module. *“...Where do I attach the ECG leads?...”* was the most asked question while wearing the device. The rest of the participants were able to complete the task without any complication, showing the ease of use of the application.

#### III) Satisfaction

**Fig. 19:**
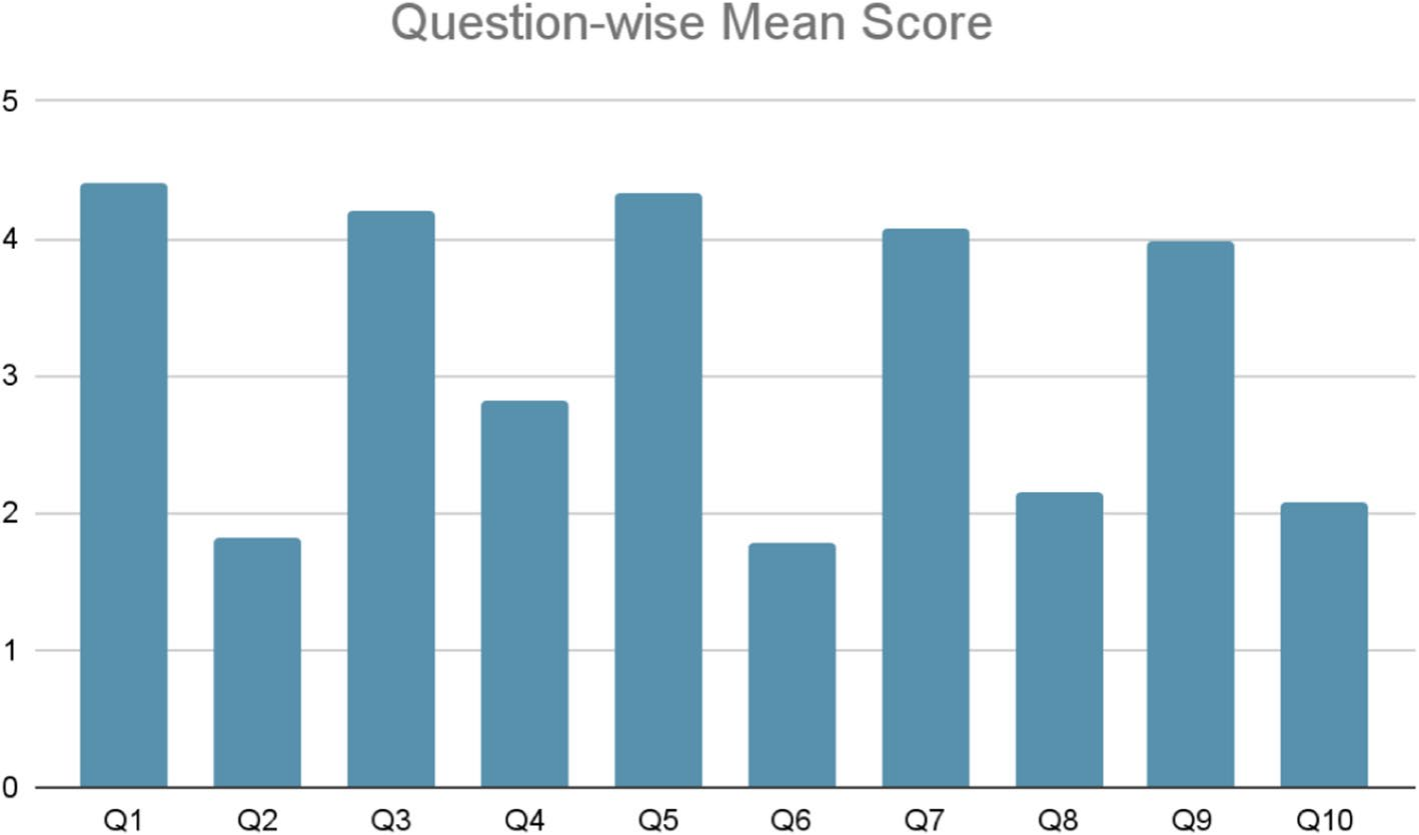
System Usability Score (SUS) of the application

System Usability Scale (SUS) is used as another indicator for the evaluation purpose. SUS score, following Brooke’s [40] evaluation technique, is a quick and reliable metric while measuring the usability of a product or a system based on user feedback collected through 10 questions with 5 responses concerning a strong agreement to strong disagreement. The set of these questions is presented in Table 6. Feedback from the same participants’ group has been collected through a survey after the usage of the system is used to evaluate the usability of the system. The system was rated good by the SUS score with an average score of 75.8125%. Figure 19 shows the question-wise mean score concluding that users find the system easy to use and they would use it again in the future.

**Table 6:** Ten items that make up the standard SUS (odd-numbered items are positively phrased, even-numbered items are negatively worded)

Serial	Question
1	I think that I would like to use this system frequently.
2	I found the system unnecessarily complex.
3	I thought the system was easy to use.
4	I think that I would need the support of a technical person to be able to use this system
5	I found the various functions in this system were well integrated.
6	I thought there was too much inconsistency in this system.
7	I would imagine that most people would learn to use this system very quickly.
8	I found the system very cumbersome to use.
9	I felt very confident using the system.
10	I needed to learn a lot of things before I could get going with this system.

Even though only 60% of the participants had adequate knowledge about mobile phones and other computational devices, 47.5% of the total participants strongly agreed to use the system frequently and 45% of them agreed to use it on a regular basis. 37.5% of the participants strongly agreed that the system was easy to use while 2.5% participants strongly agreed about the need for assistance while using Predictis. Along with these ten questions of the SUS method, participants were also asked to provide their comments concerning this system. 82.5% of participants responded with positive feedback and expressed their concerns regarding the recommendation of the system to others.

#### IV) Subjective feedback

After the test data acquisition from users is completed, feedback was obtained from all of them. A semi-structured interview was conducted with all 40 users. All the discussions were recorded with the user’s permission for better analysis of the feedback. After collecting all the video recordings, those recordings were analyzed and some key patterns were identified. The outcome of the analysis of the qualitative data is illustrated below:

- *Requirement of Training:* One of the patterns extracted from the recorded videos was that most of the participants felt they would require some training for putting the hardware device on. Mostly they need assistance for putting the leads of ECG monitor AD8232 in the right places. They also mentioned a user manual for proper usage of the Hardware system. Considering this matter, one of the end-users said, “The Application part of this system is pretty simple. However, to get proper values from sensors, you have to put the sensors in the right place. A user manual for setting up the hardware would benefit the users greatly.”
- *Cholesterol and blood sugar:* This system is mainly developed for aged people who haven’t been diagnosed with CVD yet. Aged people need to do cholesterol and blood sugar tests on a comparatively regular basis. To successfully create a profile users must know their recent cholesterol and blood sugar level. Else the system would not be able to predict the risk level of having CVD in the future. Regarding this one of the users asked, “Suppose, I don’t know my cholesterol level then I have to go to the hospital to get my cholesterol level, which will be time-consuming. What you can do is incorporate an automated system for those who don’t know their cholesterol and blood sugar levels which will book a home appointment with a nurse at a nearby hospital. And after getting the results the users can successfully complete the tests.”
- *Hardware prototype:* The hardware prototype developed using ECG monitor AD8232, is wiry. The one-lead ECG monitors used in smartwatches are compact and at the same time more accurate, but expensive as they are more integrated. Keeping this in mind, one of the users mentioned, “As an initial module the hardware device is much more compact, but it would be more convenient to set up a hardware device which is more integrated and compact. In smartwatches, you can just touch a button on the watch and the ECG gets recorded.”
- *Usefulness to aged people:* The developed system was complemented by all of the users who participated in this interview. It is convenient for aged people who haven’t been diagnosed with CVD yet. They don’t get enough time to have a heart checkup or they are not conscious enough to get a checkup as they haven’t faced any problems yet. Using this application, they can identify their risks of having CVD in the future and work accordingly to minimize the consequences. Regarding this, one of the users said, “This is a very innovative way of thinking, as we don’t get enough time in this busy life we can’t do proper medical checkups unless we face any major problems. This system will be a major help in detecting CVD in the early stage and will lessen the accidental deaths caused by strokes and heart attacks.”

## Discussion

The study introduced a system consisting of an IoT device including different biomedical sensors, an android interface for monitoring real-time sensor data, and a cloud infrastructure for processing the real-time data using ML techniques for the prediction of the CVD risk level. Comparisons with some of the existing IoT-based systems reviewed in the related works are described in detail below and summarized in the Tables 7 and 8.

**Table 7:** Comparison with related work in IoT for Healthcare

Features	Embedded platform	Prediction Level / Classes	Evaluation of System	Monitoring of Patients with emergency alerts
[23]	Client computer with IoT sensors: AD8232 ECG Monitor.	Presence/Absence of disease only	Not done	No
[25]	IoMT Based cloud infrastructure having client computers as User Interface (UI). Sensor technologies not specified.	Presence/ Absence of disease only	Not done	Monitoring only
[9]	IoT based disease diagnosis model with IoT gadgets. Sensor technologies and UI not specified.	presence and absence of heart disease only	Done using means of ten-fold cross validation	No
[10]	IoT body area network (BAN) using ECG and heart rate sensors and smart phone based Platform as UI.	Presence/Absence of disease only	Not Done	No
[24]	IoT based patient monitoring system using Pressure Sensor, Heart Rate sensor etc. UI not mentioned.	presence and absence of stroke risk only	Not Done	Yes
[30]	IoT based prediction and monitoring system having blood pressure, pulse, oximeter sensors. UI not mentioned.	Presence/Absence of disease only	Not done	Yes
**Proposed**	Android Device with Iot based wearable Integrated Device incorporating AD8232 ECG Monitor, Blood Pressure Monitor and Heart rate sensors.	Two type of prediction:	Detailed system evaluation done in terms of effectiveness, efficiency and satisfaction on both quantitative and qualitative data extracted from 40 participants	Real- time monitoring system with constant checking of anomalies in ECG, BP and Heart rate and emergency alert
		1. Presence/Absence of disease (Two zone classification)		
		2. Disease risk level determination (Three-zone classification)		

**Table 8:** Comparison with related work in IoT for Healthcare

Features	Learning Models	Training dataset	Prediction Accuracy (Testing)	ECG signal processing
[23]	Modified deep convolutional neural network	UCI heart disease dataset (303 datapoint)	Two level :93%	Not done
			Three level:-	
[25]	MSSO-ANFIS	UCI heart disease dataset	Two level :98.79%	No ECG sensor mentioned.
			Three level:-	
[9]	decision tree classification algorithm based on Iterative Dichotomiser 3 (j48 classifier)	UCI heart disease dataset	Two level :91.48%	No ECG sensor mentioned
			Three level:-	
[10]	Random Forest classifier	UCI heart disease dataset (270 datapoint)	Two level :99%	implements an edge computing technique to find out three slope characteristics of the ST wave.
			Three level:-	
[24]	Random Forest	consist of 191 records of the patient	Two level : 93%	No ECG sensor mentioned
			Three level:-	
[30]	SVM	UCI heart disease dataset	Two level :97%	No ECG sensor mentioned
			Three level:-	
**Proposed**	Stacking Classifier	UCI heart disease dataset (920 datapoint)	Two level :91%	QRS complex detection using pan-tompkins algorithm and slope measurement of the ST segment.
			Three level:80.4%	

### Embedded platforms

A handful of research [9, 10, 23–25, 30] focused on IoT-based cloud infrastructure with computer software as an interface only perceivable by physicians. Among them [9, 25] proposed a framework only, where no specific IoT sensors and any user-end interface were mentioned. The studies [23, 24, 30] mentioned using different biomedical IoT sensors but no proper user-end interface was mentioned. Only the study [10] mentioned both IoT sensors and smartphone-based platforms as user interference. However, our study suggested a complete system consisting of a compact wearable device using biomedical IoT sensors(e.g. ECG AD8232, Blood Pressure Monitor, Pulse Rate) and a user-end application developed in android perceivable not only by physicians but also by general people. The proposed embedded system has the functional ability to monitor the user, provide some medical consultation, and record the history of prediction and emergency contact in any uncertain situation, making it more useful on the user end. Thus the system provides the users with the ability to check their heart condition by staying at home without taking any medical test.

### ECG signal processing

Even though some papers [10, 23] have mentioned the use of AD8232 ECG sensor in their proposed system or IoT frameworks, the acquisition of features from the noisy ECG data relevant to the CVD prediction have not been discussed in them. Our study has thoroughly described the procedure of measuring the slope of the ST segment through the Pan-Tompkins algorithm’s QRS complexes detection.

### Monitoring of patients with emergency alerts

Some of the prior studies proposed the monitoring of high-risk patients through sensors interfaced with user-end devices. The system proposed in [25] only focused on detecting and continuously monitoring patients’ heart conditions through the predicted result of the proposed algorithm only. The papers [24, 30] also suggested monitoring patients to predict the chances of disease occurrence along with alerting doctors to take necessary action.

Our proposed system not only suggested CVD risk estimation through prediction from sensor data but also a real-time Mobile Health care-based monitoring system by constantly checking vital signs and providing an immediate alert to the caregiver in case of emergency. Additionally, the system also provides with preserving the data so that patient conditions can easily be understood through graphical visualization of data.

### Prediction level / classes

Most of the studies in CVD prediction only focused on detecting the disease only, simply limiting the prediction classes to the presence and absence of disease. The system that we have proposed has further expanded to predicting the risk level of having CVD in the parameters of high-risk, moderate risk and low-risk levels. The users estimated to be having a moderate risk of CVD can keep their condition in check through regular monitoring, ample exercise, rest and diet suggested by physicians.

### Learning models, dataset, accuracy

Studies related to Cardio-Vascular diseases prediction have explored a wide variety of classification algorithms mostly in the UCI dataset. Our study has been conducted on the same dataset but with limited features. Since we focused on building an automated system the features which could not be accumulated from sensor data or need expensive medical diagnostics have not been included in the training dataset, which may have affected our prediction accuracy from the state of the art. However, the studies mentioned in Table 7 have mostly focused on finding the best-performing algorithm on the complete dataset rather than building an automated system. Thus their accuracy ranges from 93 to 99% which is comparatively better than ours.

While most of the works [9, 10, 24, 30] focused on conventional ML algorithms, some introduced hybrid and ensemble-based classifiers. The study [23] proposed a Modified deep convolutional neural network as the best-performing algorithm. Another study [25], proposed modified salp swarm optimization (MSSO) and an adaptive neuro-fuzzy inference system (ANFIS). Our proposed system explored the prediction of cardio-vascular diseases by experimenting with ten different models of conventional ML algorithms. Our system was built using a stacking classifier model with an f1-value of 91% in two levels and 80.4% in three levels. Recall /Sensitivity is the approach that identifies the people with cardio-vascular diseases (true positive rate), finding how efficiently the model can correctly determine the patients who have the chance of heart disease. The proposed model shows 91% recall for two-level and 79.3% recall for three-level classification respectively.

Additionally, in most of the studies, testing and trial data are separated and the accuracy of the performance of the ML algorithm is measured. No further evaluation of the system study has been done in any of the works. Our study demonstrated an evaluation study for performance evaluation and the usability of the integrated system in terms of effectiveness, efficiency and satisfaction through the participation of 40 participants. For evaluating effectiveness, the performance of the deduced algorithm is tested by feeding a real dataset to the system through an app and finding a prediction result. In the efficiency study, the quality and state of the system have been evaluated on certain parameters. The system Usability Scale is taken as an indicator for system satisfaction evaluation. Additionally, by conducting a study with end-users, the usability feedback was taken and the result of the whole system was evaluated.

## Conclusion

The goal of the study was to introduce a complete system with an efficient wearable device and user interface for end users to be able to predict risk levels of cardio-vascular diseases by monitoring real-time data. For the successful prediction task, the study explored the prediction of cardio-vascular diseases by experimenting with 11 different models of ML algorithms and practical use of the hardware to collect real-time data at the user level. Two stacking classifier models with an f1-value of 91 percent in two levels and 80.4 percent in three levels were used to develop our prediction system. For two zone classification, the constructed stacking classifier has a precision and recall score of 91%, and for three zone classification, these values are 81.6% and 79.3% respectively. Finally, an evaluation of the system was conducted to determine the efficiency, effectiveness and usability of the developed system from the real-time data of 40 participants. For measuring effectiveness, the system’s ability to give accurate prediction is evaluated from these participant data and the results showed an f1 score of 89.3 and 87.7 for two and three-zone, respectively. For efficiency, the system’s quality and state while predicting have been evaluated and the results showed that 19.325 clicks and 80.21 seconds are needed on average to complete the task. The System Usability Scale (SUS) is employed as the indicator to measure usability and the resultant score of 75.8125 is viewed as a good metric for usability.

Compared to the previous researches, this study has several advantages. Previous research was conducted on heart disease prediction with neural networks and conventional ML techniques. Most of the research [18–21] focused on finding the best performing algorithm for predicting heart disease. Some of the prior studies [9, 18] did not adopt any sensors or IoT systems for collecting real-time data, while some studies [23] collected data using these sensor technologies. However, our study included ECG sensor data, blood pressure sensor data, and pulse sensor for predicting CVD, and a compact wearable device was prepared for collecting real-time data from the user. The disease prediction is made using the user’s profile data and real-time data providing effective disease detection. Users can check their heart condition by staying at home without taking any medical tests.

Our system comes with some limitations too. The system is particularly created to predict cardio-vascular diseases commonly responsible for angina or heart attack but can not detect any heart disease. The system has been evaluated by a participation group having an age range of 25 to 60 years. Moreover, some potential threats to validity can occur in the system if the medical data collected from the user is aged more than a few months, as medical condition of the user can change during this gap. Also, if the wearable device isn’t attached properly, noisy data can generate an erroneous prediction. As the hardware device is made out of a microcontroller and different biomedical sensors, it is not that compact yet.

This system can be expanded in the future for other diseases, as well as heart attacks, by adding more features to the model. In addition, the hardware device can be made more light weighted and compact for the users in the future. Furthermore, using the dataset of patients of a particular country or region will increase the accuracy of prediction for the people living there, which can broaden the area of our work as well.

## Data Availability

The UCI dataset, generated and analyzed during the current study are publicly available at https://archive.ics.uci.edu/ml/datasets/heart+disease.
